# Crystal structure of the *cis* and *trans* polymorphs of bis­[μ-2-(1,3-benzo­thia­zol-2-yl)phenolato]-κ^3^ *N*,*O*:*O*;κ^3^ *O*:*N*,*O*-bis­[*fac*-tri­carbonyl­rhenium(I)]^1^

**DOI:** 10.1107/S2056989017001347

**Published:** 2017-01-31

**Authors:** Maruthupandiyan Priyatharsini, Bhaskaran Shankar, Malaichamy Sathiyendiran, Navaneethakrishnan Srinivasan, Rajaputi Venkatraman Krishnakumar

**Affiliations:** a Department of Physics, Thiagarajar College, Madurai 625 009, Tamil Nadu, India; b Department of Chemistry, University of Hyderabad, South Campus, Hyderabad 500 046, Telengana, India

**Keywords:** crystal structure, rhenium tricarbonyl complexes, polymorphism

## Abstract

The title dinuclear complex, [Re_2_(C_13_H_8_NOS)_2_(CO)_6_], crystallizes in two polymorphs where the 2-(1,3-benzo­thia­zol-2-yl)phenolate ligands and two carbonyl groups are *trans*- (**I**) or *cis*-arranged (**II**) with respect to the [Re_2_O_2_(CO)_4_] core. Polymorphs **I** and **II** exhibit a crystallographically imposed centre of symmetry and a twofold rotation axis, respectively. The structures may be described as being formed by two octa­hedrally distorted metal-coordinating units fused through μ-oxido bridges, leading to edge-sharing dimers. The crystal packing is governed by C—H⋯O hydrogen-bonding inter­actions, forming chains parallel to the *c* axis in **I** and a three-dimensional network in **II**.

## Abstract 2

The mol­ecular structure of the title compounds may be visualized as two octa­hedral metal coordinated units, fused through μ-oxide bridges across symmetry centres, leading to edge-sharing dimers.

## Chemical context   

Organometallic complexes are regarded as inter­esting and important compounds owing to their versatile photophysical, photochemical and biological properties. In particular, the importance of the use of metal complexes in medicine began with the discovery of the anti-cancer activity of *cis*-platin (Rosenberg *et al.*, 1965 ▸). Since then, attempts to synthesize and characterize novel organometallics with potential pharmaceutical applications remains the main focus of anti­cancer drug discovery.

While it has been discovered recently that some rhenium–indolato complexes exhibit light-induced anti-cancer activity (Kastl *et al.*, 2013 ▸), a number of tricarbon­yl–rhenium complexes are well known agents in the field of biomedical imaging (Lo *et al.*, 2010 ▸, 2011 ▸). Several rhenium(I) tricarbonyl heterocyclic complexes are known to exhibit intense luminescence in the visible region and, owing to their stability to photodecomposition, are promising candidates for solar energy conversion applications (Wallace & Rillema, 1993 ▸). In the context of earlier works (Shi *et al.*, 1996 ▸; Bradshaw & Westwell, 2004 ▸; Potgieter *et al.*, 2012 ▸) suggesting benzo­thia­zole derivatives to be promising ligands for rhenium which possess potential usefulness in radiotherapy, the intra- and inter­molecular features of the crystal structures of the title compound may well be regarded as relevant. More recently, a host of rhenium–tricarbonyl complexes containing heterocyclic derivatives have been shown to exhibit anti­microbial properties (Kumar *et al.*, 2016 ▸). In a recent review, a systematic evaluation of neutral Re^I^ tricarbonyl complexes was undertaken for their suitability as organic light-emitting diodes (Zhao *et al.*, 2016 ▸).
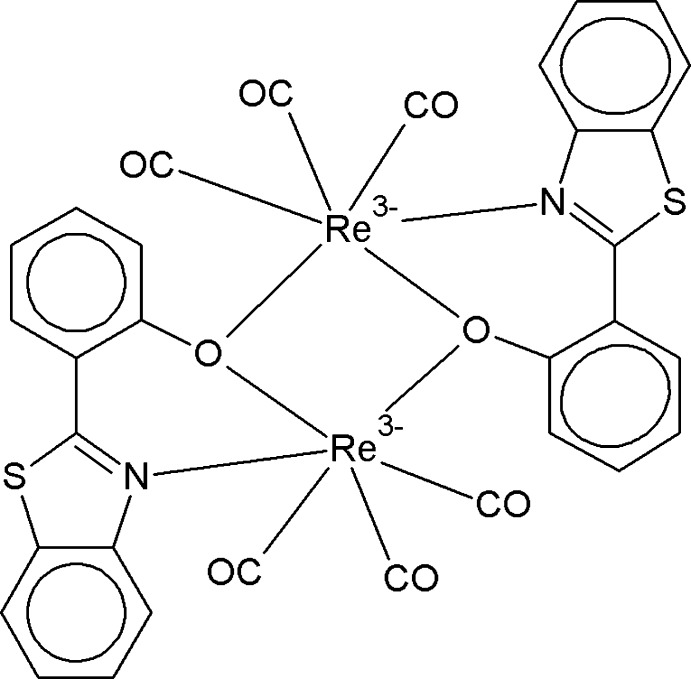


## Structural commentary   

**Figure 1:**
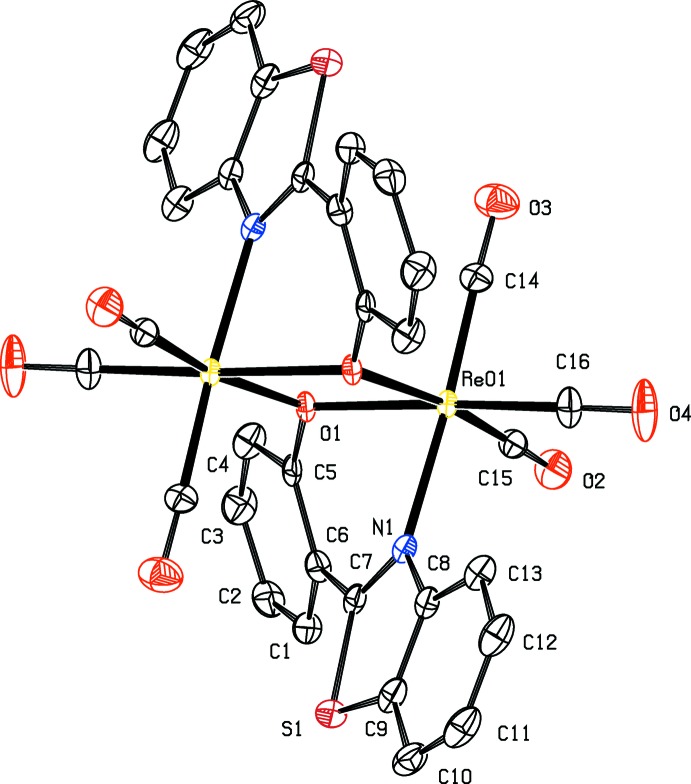
The mol­ecular structure of **I**, with displacement ellipsoids drawn at the 50% probability level. Unlabelled atoms are related to labelled atoms by (1 − *x*, 1 − *y*, 1 − *z*). H atoms have been omitted for clarity.

**Figure 2:**
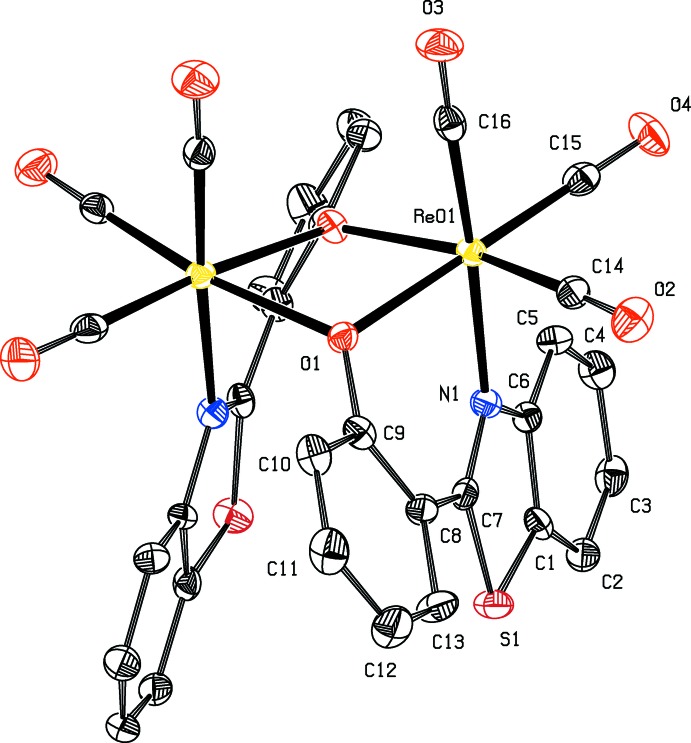
The mol­ecular structure of **II**, with displacement ellipsoids drawn at the 50% probability level. Unlabelled atoms are related to labelled atoms by (−*x*, *y*, 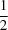 − *z*). H atoms have been omitted for clarity.

The title compound, [Re(CO)_3_(*L*)]_2_ where *L*= 2-(1,3-benzo­thia­zol-2-yl)phenolate, crystallizes in two different forms, *viz*. the *trans* form (**I**, Fig. 1 ▸) in the triclinic space group *P* 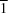 and the *cis* form (**II**, Fig. 2 ▸) in the ortho­rhom­bic space group *Pbca*. The structure of the compound may be described as being composed of two octa­hedral metal-coordinating units fused through μ-oxido bridges leading to edge-sharing dimers. The presence of the inversion centre in **I** leads to Re—O-bridged centrosymmetric dimeric mol­ecular units. In **II**, dimerization through Re—O bridging is achieved through a twofold rotation axis. In both **I** and **II**, coordination around the rhenium atom is similar, the metal exhibiting a distorted octa­hedral environment with atoms C16 and N1 occupying the apical sites and atoms C14, C15, O1 and O1^i^/O1^ii^ at the equatorial plane [symmetry codes: (i) 1 − *x*, 1 − *y*, 1 − *z*; (ii) −*x*, *y*, 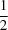 − *z*]. The N1—Re01—O1^i^—C9^i^ torsion angle associated with the Re—O bridging of symmetry-related mol­ecules in *trans* polymorph **I** [137.1 (5)°] is distinctly different from the corresponding value in the *cis* polymorph **II** [−59.4 (3)°]. The Re⋯Re and O1⋯O1 separations in the Re_2_O_2_ core are 3.4799 (5) and 2.581 (8) Å, and 3.4332 (5) and 2.535 (4) Å in **I** and **II**, respectively.

The conformation of the ligand in **I** and **II** is significantly different. The dihedral angles between the planar benzo­thia­zole unit and the benzene rings in **I** and **II** are 32.23 (18) and 22.78 (8)°, respectively. The value observed in **II** closely agrees with that observed in the crystal structure of 2-(4-hy­droxy­phen­yl)benzo­thia­zole [18.49 (6)°; Teo *et al.*, 1995 ▸], which inter­estingly crystallizes in the same space group. The larger value observed in **I** may be attributed to the ‘flipping’ of the twofold symmetry into an inversion centre.

## Supra­molecular features   

**Figure 3:**
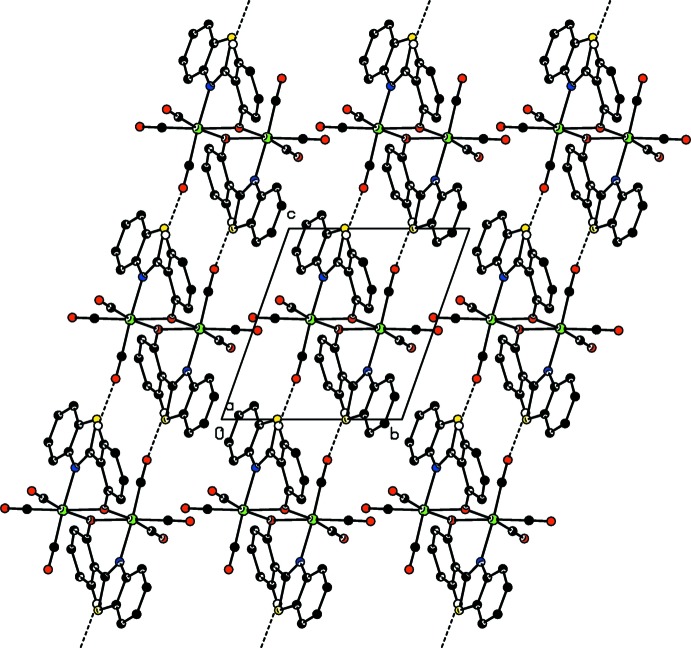
Crystal packing of **I**, showing the formation of mol­ecular chains parallel to the *c* axis *via* C—H⋯O hydrogen bonds (dashed lines). H atoms not involved in hydrogen bonding are omitted.

The crystal structures of **I** and **II** are governed by C—H⋯O hydrogen bonds which significantly differ in their strengths and the mode of participation of the carbonyl O atoms. In **I**, the O3 atom of the apical carbonyl group C16=O3 plays a role in connecting the mol­ecules across inversion centres into a chain along the *c* axis (Fig. 3 ▸, Table 1 ▸). In addition, a short O4⋯O4^iii^ contact [symmetry code: (iii) –*x* + 1, –*y* + 2, –*z* + 1] involving centrosymmetrcally related carbonyl groups C15=O4 [2.792 (10) Å] is present, linking the chains along the *b* axis to form layers parallel to the *bc* plane.

**Figure 4:**
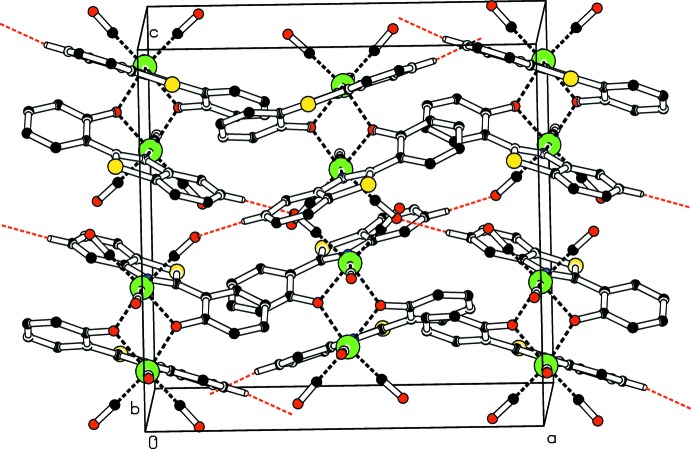
Crystal packing of **II** approximately, viewed along the *b* axis, showing mol­ecules linked into a the three-dimensional network through C—H⋯O hydrogen bonds (red dashed lines). H atoms not involved in hydrogen bonding are omitted.

In **II**, the oxygen atom of the equatorial carbonyl group C14=O2 links the mol­ecules across the glide planes into a three-dimensional network (Fig. 4 ▸, Table 2 ▸). Similarly to that observed in **I**, a C—H⋯O hydrogen bond involving the O3 atom of the apical carbonyl group C16=O3 is present, which extends along the *b* axis through translation. Therefore it may be concluded that in both the *trans* and *cis* polymorphs, the mode of participation to the hydrogen-bonding network of the O atom of the apical carbonyl group is through simple translation, while there is a significant ‘switching’ in the choice of the O atoms of the equatorial carbonyl groups. A common feature between the two structures is that one of the three carbonyl groups, namely C14=O2 in **I** and C15=O4 in **II**, forbids its O atom from participating in the inter­molecular inter­actions.

## Database survey   

A search in the Cambridge structural Database (Version 5.35, November 2014 update; Groom *et al.*, 2016 ▸) for μ-oxido bridging dinuclear complexes of rhenium having an octa­hedral coordination environment similar to that observed in the title compounds (*i.e.* involving three carbonyl C atoms, two oxygens and a nitro­gen) was made. The search returned 45 crystal structures with three-dimensional coordinates determined, excluding duplicate structure determinations and having an *R* factor less than 0.075. Out of these 45 crystal structures, 25 crystallize in the monoclinic, nine in the triclinic, eight in the ortho­rhom­bic and three in the trigonal systems. In these compounds, the Re⋯Re distance ranges from 3.330 to 3.501 Å, the O⋯O separation within the Re_2_O_2_ core ranges from 2.485 to 2.701 Å, and Re—O bond lengths from 2.065 to 2.215 Å.

## Synthesis and crystallization   

For **I**:

A mixture of Re_2_(CO)_10_ (101.3 mg, 0.1552 mmol), 2-(1,3-benzo­thia­zol-2-yl)phenol (69.7 mg, 0.307 mmol) and 2-phenyl-2-imidazoline (45.8 mg, 0.323 mmol) in toluene (10 ml) in a Teflon flask was placed in a steel bomb. The bomb was placed in an oven maintained at 433 K for 48 h and then cooled to 298 K. Pale-yellow crystals were obtained and separated by filtration.

For **II**:

A mixture of Re_2_(CO)_10_ (101.8 mg, 0.156 mmol), 2-(1,3-benzo­thia­zol-2-yl)phenol (69.9 mg, 0.308 mmol) and 2-(pyrid­in-4-yl)-1-(2,4,6-tri­methyl­benz­yl)-1*H*-benzo[*d*]imidazole (101.1 mg, 0.309 mmol) in toluene (10 ml) in a Teflon flask was placed in a steel bomb. The bomb was placed in an oven maintained at 433 K for 48 h and then cooled to 298 K. Pale-yellow crystals were obtained and separated by filtration.

## Refinement   

Crystal data, data collection and structure refinement details are summarized in Table 3 ▸. In both **I** and **II**, the H atoms were placed in calculated positions (C—H = 0.93–0.97 Å) and were included in the refinement in the riding-model approximation, with *U*_iso_(H) set at 1.2–1.5*U*_eq_(C). In **I**, two outliers (9 11 2, 2 2 4) were omitted in the last cycles of refinement.

## Supplementary Material

Crystal structure: contains datablock(s) I, II. DOI: 10.1107/S2056989017001347/rz5204sup1.cif

Structure factors: contains datablock(s) I. DOI: 10.1107/S2056989017001347/rz5204Isup6.hkl

Structure factors: contains datablock(s) II. DOI: 10.1107/S2056989017001347/rz5204IIsup7.hkl

CCDC references: 1529701, 1529700

Additional supporting information:  crystallographic information; 3D view; checkCIF report

## Figures and Tables

**Table 1:** Hydrogen-bond geometry (Å, °) for **I** [Chem scheme1]

*D*—H⋯*A*	*D*—H	H⋯*A*	*D*⋯*A*	*D*—H⋯*A*
C13—H13⋯O3^i^	0.95	2.52	3.276 (8)	137

Symmetry code: (i) 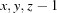.

**Table 2:** Hydrogen-bond geometry (Å, °) for **II** [Chem scheme1]

*D*—H⋯*A*	*D*—H	H⋯*A*	*D*⋯*A*	*D*—H⋯*A*
C4—H4⋯O2^i^	0.93	2.49	3.387 (4)	163
C2—H2⋯O3^ii^	0.93	2.64	3.467 (5)	149

Symmetry codes: (i) 
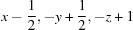
; (ii) 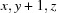.

**Table 3:** Experimental details

	**I**	**II**
Crystal data		
Chemical formula	[Re_2_(C_13_H_8_NOS)_2_(CO)_6_]	[Re_2_(C_13_H_8_NOS)_2_(CO)_6_]
*M*_r_	992.99	992.99
Crystal system, space group	Triclinic, *P* 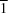	Orthorhombic, *P* *b* *c* *n*
Temperature (K)	100	296
*a*, *b*, *c* (Å)	8.9250 (11), 9.7342 (12), 10.0844 (12)	16.1480 (7), 11.6519 (5), 15.6329 (8)
α, β, γ (°)	66.438 (5), 75.636 (5), 63.585 (5)	90, 90, 90
*V* (Å^3^)	716.59 (16)	2941.4 (2)
*Z*	1	4
Radiation type	Mo *K*α	Mo *K*α
μ (mm^−1^)	8.64	8.42
Crystal size (mm)	0.28 × 0.18 × 0.15	0.25 × 0.18 × 0.12

Data collection		
Diffractometer	Bruker SMART APEX CCD	Bruker SMART APEX CCD
Absorption correction	Multi-scan (*SADABS*; Bruker, 2009 ▸)	Multi-scan (*SADABS*; Bruker, 2009 ▸)
*T*_min_, *T*_max_	0.168, 0.357	0.18, 0.38
No. of measured, independent and observed [*I* > 2σ(*I*)] reflections	23724, 3325, 3113	11735, 3510, 2943
*R*_int_	0.105	0.027
(sin θ/λ)_max_ (Å^−1^)	0.654	0.688

Refinement		
*R*[*F* ^2^ > 2σ(*F* ^2^)], *wR*(*F* ^2^), *S*	0.036, 0.088, 1.08	0.027, 0.060, 1.07
No. of reflections	3325	3510
No. of parameters	208	208
H-atom treatment	H-atom parameters constrained	H-atom parameters constrained
Δρ_max_, Δρ_min_ (e Å^−3^)	2.91, −2.74	1.09, −0.95

Computer programs: *APEX2* and *SAINT* (Bruker, 2009 ▸), *SHELXS2013* (Sheldrick, 2008 ▸), *SHELXL2014* (Sheldrick, 2015 ▸), *PLUTON* (Spek, 2009 ▸) and *publCIF* (Westrip, 2010 ▸).

## supplementary crystallographic information

### (I) trans-Bis[µ-2-(1,3-benzothiazol-2-yl)phenolato]-κ3N,O:O;κ3O:N,O-bis[fac-tricarbonylrhenium(I)] Crystal data

**Table:**

[Re_2_(C_13_H_8_NOS)_2_(CO)_6_]	*Z* = 1
*M**_r_* = 992.99	*F*(000) = 468
Triclinic, *P*1	*D*_x_ = 2.301 Mg m^−^^3^
*a* = 8.9250 (11) Å	Mo *K*α radiation, λ = 0.71073 Å
*b* = 9.7342 (12) Å	Cell parameters from 3113 reflections
*c* = 10.0844 (12) Å	θ = 2.5–27.7°
α = 66.438 (5)°	µ = 8.64 mm^−^^1^
β = 75.636 (5)°	*T* = 100 K
γ = 63.585 (5)°	Needle, yellow
*V* = 716.59 (16) Å^3^	0.28 × 0.18 × 0.15 mm

### (I) trans-Bis[µ-2-(1,3-benzothiazol-2-yl)phenolato]-κ3N,O:O;κ3O:N,O-bis[fac-tricarbonylrhenium(I)] Data collection

**Table:**

Bruker SMART APEX CCD diffractometer	3113 reflections with *I* > 2σ(*I*)
ω scans	*R*_int_ = 0.105
Absorption correction: multi-scan (SADABS; Bruker, 2009)	θ_max_ = 27.7°, θ_min_ = 2.5°
*T*_min_ = 0.168, *T*_max_ = 0.357	*h* = −11→11
23724 measured reflections	*k* = −12→12
3325 independent reflections	*l* = −13→13

### (I) trans-Bis[µ-2-(1,3-benzothiazol-2-yl)phenolato]-κ3N,O:O;κ3O:N,O-bis[fac-tricarbonylrhenium(I)] Refinement

**Table:**

Refinement on *F*^2^	0 restraints
Least-squares matrix: full	Hydrogen site location: inferred from neighbouring sites
*R*[*F*^2^ > 2σ(*F*^2^)] = 0.036	H-atom parameters constrained
*wR*(*F*^2^) = 0.088	*w* = 1/[σ^2^(*F*_o_^2^) + (0.0558*P*)^2^] where *P* = (*F*_o_^2^ + 2*F*_c_^2^)/3
*S* = 1.08	(Δ/σ)_max_ = 0.001
3325 reflections	Δρ_max_ = 2.91 e Å^−^^3^
208 parameters	Δρ_min_ = −2.74 e Å^−^^3^

### (I) trans-Bis[µ-2-(1,3-benzothiazol-2-yl)phenolato]-κ3N,O:O;κ3O:N,O-bis[fac-tricarbonylrhenium(I)] Special details

**Table:**

Geometry. All esds (except the esd in the dihedral angle between two l.s. planes) are estimated using the full covariance matrix. The cell esds are taken into account individually in the estimation of esds in distances, angles and torsion angles; correlations between esds in cell parameters are only used when they are defined by crystal symmetry. An approximate (isotropic) treatment of cell esds is used for estimating esds involving l.s. planes.
Refinement. Refined as a 2-component perfect inversion twin.

### (I) trans-Bis[µ-2-(1,3-benzothiazol-2-yl)phenolato]-κ3N,O:O;κ3O:N,O-bis[fac-tricarbonylrhenium(I)] Fractional atomic coordinates and isotropic or equivalent isotropic displacement parameters (Å^2^)

**Table:**

	*x*	*y*	*z*	*U*_iso_*/*U*_eq_	
Re01	0.36347 (2)	0.70142 (2)	0.47571 (2)	0.01179 (10)	
S1	0.5151 (2)	0.69074 (18)	−0.00066 (15)	0.0204 (3)	
O1	0.3841 (5)	0.4733 (5)	0.4716 (4)	0.0136 (7)	
O2	0.0115 (6)	0.9108 (6)	0.3751 (5)	0.0285 (11)	
O3	0.2004 (7)	0.6662 (7)	0.7869 (5)	0.0329 (12)	
O4	0.3450 (7)	1.0258 (6)	0.4663 (8)	0.0413 (14)	
N1	0.4733 (6)	0.7154 (5)	0.2528 (5)	0.0143 (9)	
C1	0.6280 (7)	0.7795 (7)	0.0280 (6)	0.0206 (12)	
C2	0.7395 (9)	0.8452 (8)	−0.0704 (7)	0.0258 (14)	
H2	0.7625	0.8448	−0.1674	0.031*	
C3	0.8153 (8)	0.9111 (8)	−0.0215 (8)	0.0283 (15)	
H3	0.8930	0.9550	−0.0856	0.034*	
C4	0.7797 (8)	0.9142 (8)	0.1200 (8)	0.0267 (14)	
H4	0.8337	0.9599	0.1507	0.032*	
C5	0.6676 (8)	0.8521 (7)	0.2161 (7)	0.0201 (12)	
H5	0.6427	0.8563	0.3120	0.024*	
C6	0.5912 (7)	0.7828 (6)	0.1703 (6)	0.0164 (11)	
C7	0.4244 (7)	0.6635 (6)	0.1754 (6)	0.0158 (10)	
C8	0.3038 (7)	0.5852 (7)	0.2262 (6)	0.0173 (11)	
C9	0.2952 (7)	0.4856 (6)	0.3742 (6)	0.0144 (10)	
C10	0.1877 (8)	0.4040 (7)	0.4184 (7)	0.0214 (12)	
H10	0.1813	0.3356	0.5164	0.026*	
C11	0.0890 (9)	0.4215 (8)	0.3198 (7)	0.0255 (13)	
H11	0.0175	0.3636	0.3513	0.031*	
C12	0.0942 (8)	0.5229 (8)	0.1760 (7)	0.0225 (12)	
H12	0.0248	0.5369	0.1101	0.027*	
C13	0.2023 (8)	0.6028 (7)	0.1310 (6)	0.0208 (12)	
H13	0.2073	0.6714	0.0329	0.025*	
C14	0.1446 (8)	0.8311 (7)	0.4146 (6)	0.0203 (12)	
C15	0.3586 (8)	0.9012 (7)	0.4703 (7)	0.0244 (13)	
C16	0.2666 (8)	0.6763 (7)	0.6697 (6)	0.0176 (11)	

### (I) trans-Bis[µ-2-(1,3-benzothiazol-2-yl)phenolato]-κ3N,O:O;κ3O:N,O-bis[fac-tricarbonylrhenium(I)] Atomic displacement parameters (Å^2^)

**Table:**

	*U*^11^	*U*^22^	*U*^33^	*U*^12^	*U*^13^	*U*^23^
Re01	0.01088 (13)	0.00927 (12)	0.01551 (13)	−0.00340 (8)	−0.00144 (7)	−0.00480 (8)
S1	0.0232 (7)	0.0199 (7)	0.0161 (6)	−0.0080 (6)	0.0009 (5)	−0.0060 (5)
O1	0.0121 (18)	0.0103 (17)	0.0185 (17)	−0.0032 (14)	0.0007 (14)	−0.0074 (14)
O2	0.016 (2)	0.029 (2)	0.032 (2)	0.0011 (18)	−0.0068 (18)	−0.0100 (19)
O3	0.027 (3)	0.042 (3)	0.025 (2)	−0.008 (2)	−0.0042 (19)	−0.012 (2)
O4	0.030 (3)	0.021 (2)	0.083 (4)	−0.010 (2)	−0.001 (3)	−0.029 (3)
N1	0.014 (2)	0.010 (2)	0.017 (2)	−0.0043 (17)	−0.0020 (17)	−0.0023 (16)
C1	0.016 (3)	0.013 (2)	0.024 (3)	−0.004 (2)	−0.001 (2)	−0.001 (2)
C2	0.024 (3)	0.020 (3)	0.022 (3)	−0.007 (2)	0.002 (2)	0.000 (2)
C3	0.016 (3)	0.021 (3)	0.035 (3)	−0.009 (2)	0.002 (2)	0.003 (2)
C4	0.020 (3)	0.020 (3)	0.036 (3)	−0.010 (2)	−0.005 (3)	−0.001 (2)
C5	0.015 (3)	0.017 (3)	0.025 (3)	−0.007 (2)	−0.003 (2)	−0.003 (2)
C6	0.014 (3)	0.011 (2)	0.021 (2)	−0.004 (2)	0.0010 (19)	−0.0040 (19)
C7	0.013 (3)	0.010 (2)	0.019 (2)	−0.0011 (19)	−0.0009 (19)	−0.0040 (19)
C8	0.015 (3)	0.016 (2)	0.020 (2)	−0.005 (2)	0.001 (2)	−0.009 (2)
C9	0.012 (2)	0.009 (2)	0.021 (2)	−0.0012 (19)	−0.0004 (19)	−0.0073 (19)
C10	0.018 (3)	0.019 (3)	0.027 (3)	−0.010 (2)	−0.004 (2)	−0.004 (2)
C11	0.026 (3)	0.025 (3)	0.032 (3)	−0.014 (3)	−0.008 (3)	−0.009 (2)
C12	0.022 (3)	0.024 (3)	0.028 (3)	−0.007 (2)	−0.009 (2)	−0.013 (2)
C13	0.020 (3)	0.018 (3)	0.020 (2)	−0.003 (2)	−0.003 (2)	−0.007 (2)
C14	0.028 (3)	0.017 (3)	0.017 (2)	−0.011 (2)	0.002 (2)	−0.006 (2)
C15	0.021 (3)	0.015 (3)	0.036 (3)	−0.004 (2)	0.000 (2)	−0.012 (2)
C16	0.014 (3)	0.022 (3)	0.018 (2)	−0.005 (2)	−0.006 (2)	−0.006 (2)

### (I) trans-Bis[µ-2-(1,3-benzothiazol-2-yl)phenolato]-κ3N,O:O;κ3O:N,O-bis[fac-tricarbonylrhenium(I)] Geometric parameters (Å, º)

**Table:**

Re01—C14	1.890 (7)	C2—H2	0.9500
Re01—C15	1.905 (7)	C3—C4	1.392 (11)
Re01—C16	1.898 (6)	C3—H3	0.9500
Re01—O1	2.162 (4)	C4—C5	1.376 (8)
Re01—O1^i^	2.171 (4)	C4—H4	0.9500
Re01—N1	2.194 (5)	C5—C6	1.396 (9)
S1—C1	1.722 (7)	C5—H5	0.9500
S1—C7	1.726 (6)	C7—C8	1.465 (8)
O1—C9	1.348 (7)	C8—C13	1.390 (9)
O1—Re01^i^	2.171 (4)	C8—C9	1.424 (7)
O2—C14	1.157 (8)	C9—C10	1.390 (8)
O3—C16	1.170 (8)	C10—C11	1.400 (9)
O4—C15	1.148 (9)	C10—H10	0.9500
N1—C7	1.317 (8)	C11—C12	1.394 (9)
N1—C6	1.405 (7)	C11—H11	0.9500
C1—C2	1.398 (8)	C12—C13	1.382 (9)
C1—C6	1.400 (8)	C12—H12	0.9500
C2—C3	1.384 (11)	C13—H13	0.9500
			
C14—Re01—C15	83.9 (3)	C5—C4—H4	119.4
C14—Re01—C16	87.9 (2)	C3—C4—H4	119.4
C15—Re01—C16	86.4 (3)	C4—C5—C6	118.8 (6)
C14—Re01—O1	98.2 (2)	C4—C5—H5	120.6
C15—Re01—O1	176.1 (2)	C6—C5—H5	120.6
C16—Re01—O1	97.0 (2)	C5—C6—C1	119.8 (5)
C14—Re01—O1^i^	170.6 (2)	C5—C6—N1	126.5 (5)
C15—Re01—O1^i^	104.5 (2)	C1—C6—N1	113.7 (5)
C16—Re01—O1^i^	96.8 (2)	N1—C7—C8	125.9 (5)
O1—Re01—O1^i^	73.12 (18)	N1—C7—S1	115.5 (4)
C14—Re01—N1	92.1 (2)	C8—C7—S1	118.6 (5)
C15—Re01—N1	97.5 (2)	C13—C8—C9	119.9 (5)
C16—Re01—N1	176.0 (2)	C13—C8—C7	120.9 (5)
O1—Re01—N1	79.11 (16)	C9—C8—C7	119.1 (5)
O1^i^—Re01—N1	82.68 (16)	O1—C9—C10	120.0 (5)
C1—S1—C7	89.8 (3)	O1—C9—C8	121.6 (5)
C9—O1—Re01	115.5 (3)	C10—C9—C8	118.3 (6)
C9—O1—Re01^i^	126.8 (3)	C9—C10—C11	120.6 (5)
Re01—O1—Re01^i^	106.88 (18)	C9—C10—H10	119.7
C7—N1—C6	110.7 (5)	C11—C10—H10	119.7
C7—N1—Re01	120.7 (4)	C12—C11—C10	120.8 (6)
C6—N1—Re01	128.5 (4)	C12—C11—H11	119.6
C2—C1—C6	121.4 (6)	C10—C11—H11	119.6
C2—C1—S1	128.4 (5)	C13—C12—C11	118.8 (6)
C6—C1—S1	110.2 (4)	C13—C12—H12	120.6
C3—C2—C1	117.7 (6)	C11—C12—H12	120.6
C3—C2—H2	121.2	C12—C13—C8	121.4 (5)
C1—C2—H2	121.2	C12—C13—H13	119.3
C2—C3—C4	121.2 (6)	C8—C13—H13	119.3
C2—C3—H3	119.4	O2—C14—Re01	179.0 (5)
C4—C3—H3	119.4	O4—C15—Re01	175.7 (6)
C5—C4—C3	121.2 (7)	O3—C16—Re01	177.0 (5)
			
C7—S1—C1—C2	−178.2 (6)	C1—S1—C7—N1	−0.3 (5)
C7—S1—C1—C6	0.3 (4)	C1—S1—C7—C8	−179.6 (4)
C6—C1—C2—C3	1.3 (9)	N1—C7—C8—C13	149.4 (6)
S1—C1—C2—C3	179.7 (5)	S1—C7—C8—C13	−31.3 (7)
C1—C2—C3—C4	−1.0 (9)	N1—C7—C8—C9	−32.5 (8)
C2—C3—C4—C5	−0.1 (10)	S1—C7—C8—C9	146.7 (4)
C3—C4—C5—C6	1.0 (9)	Re01—O1—C9—C10	−128.2 (5)
C4—C5—C6—C1	−0.8 (9)	Re01^i^—O1—C9—C10	92.4 (6)
C4—C5—C6—N1	−179.4 (5)	Re01—O1—C9—C8	49.8 (6)
C2—C1—C6—C5	−0.4 (8)	Re01^i^—O1—C9—C8	−89.7 (6)
S1—C1—C6—C5	−179.1 (4)	C13—C8—C9—O1	−175.9 (5)
C2—C1—C6—N1	178.4 (5)	C7—C8—C9—O1	6.0 (8)
S1—C1—C6—N1	−0.3 (6)	C13—C8—C9—C10	2.0 (9)
C7—N1—C6—C5	178.8 (6)	C7—C8—C9—C10	−176.0 (5)
Re01—N1—C6—C5	2.0 (8)	O1—C9—C10—C11	177.1 (6)
C7—N1—C6—C1	0.1 (7)	C8—C9—C10—C11	−0.9 (9)
Re01—N1—C6—C1	−176.7 (4)	C9—C10—C11—C12	−1.0 (10)
C6—N1—C7—C8	179.4 (5)	C10—C11—C12—C13	1.7 (10)
Re01—N1—C7—C8	−3.5 (8)	C11—C12—C13—C8	−0.6 (10)
C6—N1—C7—S1	0.1 (6)	C9—C8—C13—C12	−1.3 (9)
Re01—N1—C7—S1	177.2 (2)	C7—C8—C13—C12	176.7 (6)

Symmetry code: (i) −*x*+1, −*y*+1, −*z*+1.

### (I) trans-Bis[µ-2-(1,3-benzothiazol-2-yl)phenolato]-κ3N,O:O;κ3O:N,O-bis[fac-tricarbonylrhenium(I)] Hydrogen-bond geometry (Å, º)

**Table:**

*D*—H···*A*	*D*—H	H···*A*	*D*···*A*	*D*—H···*A*
C13—H13···O3^ii^	0.95	2.52	3.276 (8)	137

Symmetry code: (ii) *x*, *y*, *z*−1.

### (II) cis-Bis[µ-2-(1,3-benzothiazol-2-yl)phenolato]-κ3N,O:O;κ3O:N,O-bis[fac-tricarbonylrhenium(I)] Crystal data

**Table:**

[Re_2_(C_13_H_8_NOS)_2_(CO)_6_]	*D*_x_ = 2.242 Mg m^−^^3^
*M**_r_* = 992.99	Mo *K*α radiation, λ = 0.71073 Å
Orthorhombic, *P**b**c**n*	Cell parameters from 2943 reflections
*a* = 16.1480 (7) Å	θ = 2.9–28.0°
*b* = 11.6519 (5) Å	µ = 8.42 mm^−^^1^
*c* = 15.6329 (8) Å	*T* = 296 K
*V* = 2941.4 (2) Å^3^	Needle, yellow
*Z* = 4	0.25 × 0.18 × 0.12 mm
*F*(000) = 1872	

### (II) cis-Bis[µ-2-(1,3-benzothiazol-2-yl)phenolato]-κ3N,O:O;κ3O:N,O-bis[fac-tricarbonylrhenium(I)] Data collection

**Table:**

Bruker SMART APEX CCD diffractometer	2943 reflections with *I* > 2σ(*I*)
ω scans	*R*_int_ = 0.027
Absorption correction: multi-scan (SADABS; Bruker, 2009)	θ_max_ = 29.3°, θ_min_ = 2.9°
*T*_min_ = 0.18, *T*_max_ = 0.38	*h* = −12→22
11735 measured reflections	*k* = −15→14
3510 independent reflections	*l* = −21→19

### (II) cis-Bis[µ-2-(1,3-benzothiazol-2-yl)phenolato]-κ3N,O:O;κ3O:N,O-bis[fac-tricarbonylrhenium(I)] Refinement

**Table:**

Refinement on *F*^2^	0 restraints
Least-squares matrix: full	Hydrogen site location: inferred from neighbouring sites
*R*[*F*^2^ > 2σ(*F*^2^)] = 0.027	H-atom parameters constrained
*wR*(*F*^2^) = 0.060	*w* = 1/[σ^2^(*F*_o_^2^) + (0.0263*P*)^2^] where *P* = (*F*_o_^2^ + 2*F*_c_^2^)/3
*S* = 1.07	(Δ/σ)_max_ = 0.001
3510 reflections	Δρ_max_ = 1.09 e Å^−^^3^
208 parameters	Δρ_min_ = −0.95 e Å^−^^3^

### (II) cis-Bis[µ-2-(1,3-benzothiazol-2-yl)phenolato]-κ3N,O:O;κ3O:N,O-bis[fac-tricarbonylrhenium(I)] Special details

**Table:**

Geometry. All esds (except the esd in the dihedral angle between two l.s. planes) are estimated using the full covariance matrix. The cell esds are taken into account individually in the estimation of esds in distances, angles and torsion angles; correlations between esds in cell parameters are only used when they are defined by crystal symmetry. An approximate (isotropic) treatment of cell esds is used for estimating esds involving l.s. planes.

### (II) cis-Bis[µ-2-(1,3-benzothiazol-2-yl)phenolato]-κ3N,O:O;κ3O:N,O-bis[fac-tricarbonylrhenium(I)] Fractional atomic coordinates and isotropic or equivalent isotropic displacement parameters (Å^2^)

**Table:**

	*x*	*y*	*z*	*U*_iso_*/*U*_eq_	
Re01	−0.00490 (2)	0.15435 (2)	0.35969 (2)	0.01406 (6)	
S1	0.07316 (6)	0.54284 (8)	0.35632 (6)	0.0210 (2)	
O1	0.07833 (17)	0.1789 (2)	0.25483 (14)	0.0159 (6)	
O2	0.13167 (17)	0.1285 (2)	0.49308 (18)	0.0287 (7)	
O3	−0.01421 (17)	−0.1089 (3)	0.34887 (19)	0.0293 (7)	
O4	−0.13228 (17)	0.1328 (2)	0.50382 (18)	0.0302 (7)	
N1	0.00453 (16)	0.3439 (3)	0.36089 (19)	0.0158 (8)	
C1	−0.0277 (2)	0.5344 (3)	0.3923 (2)	0.0164 (8)	
C2	−0.0769 (2)	0.6243 (3)	0.4226 (2)	0.0198 (8)	
H2	−0.0566	0.6988	0.4265	0.024*	
C3	−0.1569 (2)	0.5978 (3)	0.4467 (2)	0.0212 (8)	
H3	−0.1914	0.6555	0.4672	0.025*	
C4	−0.1866 (2)	0.4861 (3)	0.4407 (3)	0.0219 (9)	
H4	−0.2410	0.4705	0.4562	0.026*	
C5	−0.1369 (2)	0.3980 (3)	0.4121 (2)	0.0173 (8)	
H5	−0.1576	0.3237	0.4083	0.021*	
C6	−0.0555 (2)	0.4210 (3)	0.3892 (2)	0.0142 (7)	
C7	0.0748 (2)	0.3953 (3)	0.3416 (2)	0.0159 (8)	
C8	0.1503 (2)	0.3426 (3)	0.3103 (2)	0.0167 (8)	
C9	0.1499 (2)	0.2380 (3)	0.2657 (2)	0.0165 (8)	
C10	0.2230 (2)	0.1963 (3)	0.2318 (2)	0.0213 (9)	
H10	0.2227	0.1273	0.2019	0.026*	
C11	0.2959 (2)	0.2549 (4)	0.2414 (2)	0.0271 (10)	
H11	0.3443	0.2262	0.2173	0.033*	
C12	0.2980 (2)	0.3564 (3)	0.2867 (3)	0.0294 (10)	
H12	0.3478	0.3950	0.2945	0.035*	
C13	0.2258 (2)	0.4003 (3)	0.3203 (3)	0.0237 (9)	
H13	0.2272	0.4694	0.3501	0.028*	
C14	0.0788 (2)	0.1374 (3)	0.4440 (3)	0.0183 (8)	
C15	−0.0848 (2)	0.1418 (3)	0.4488 (3)	0.0197 (8)	
C16	−0.0108 (2)	−0.0097 (4)	0.3516 (2)	0.0189 (9)	

### (II) cis-Bis[µ-2-(1,3-benzothiazol-2-yl)phenolato]-κ3N,O:O;κ3O:N,O-bis[fac-tricarbonylrhenium(I)] Atomic displacement parameters (Å^2^)

**Table:**

	*U*^11^	*U*^22^	*U*^33^	*U*^12^	*U*^13^	*U*^23^
Re01	0.01466 (9)	0.01247 (9)	0.01505 (10)	0.00037 (6)	0.00097 (6)	0.00113 (6)
S1	0.0214 (5)	0.0153 (4)	0.0264 (6)	−0.0040 (4)	0.0031 (4)	−0.0005 (4)
O1	0.0149 (13)	0.0178 (12)	0.0151 (14)	−0.0016 (10)	0.0013 (10)	−0.0023 (11)
O2	0.0307 (16)	0.0265 (14)	0.0288 (18)	0.0057 (12)	−0.0083 (14)	−0.0005 (13)
O3	0.0357 (17)	0.0147 (14)	0.038 (2)	−0.0035 (12)	0.0049 (14)	0.0005 (14)
O4	0.0323 (16)	0.0318 (15)	0.0265 (18)	0.0006 (13)	0.0133 (14)	0.0065 (13)
N1	0.0161 (17)	0.0151 (18)	0.0162 (19)	−0.0012 (12)	0.0013 (13)	0.0003 (12)
C1	0.0161 (17)	0.0199 (19)	0.0132 (19)	−0.0010 (15)	0.0031 (15)	0.0030 (17)
C2	0.028 (2)	0.0132 (17)	0.018 (2)	0.0005 (15)	−0.0005 (18)	0.0004 (16)
C3	0.027 (2)	0.0184 (19)	0.018 (2)	0.0071 (16)	−0.0001 (17)	−0.0029 (17)
C4	0.0179 (18)	0.025 (2)	0.022 (2)	0.0023 (16)	0.0024 (17)	0.0004 (17)
C5	0.0166 (18)	0.0140 (17)	0.021 (2)	−0.0012 (14)	−0.0008 (16)	−0.0004 (16)
C6	0.0168 (18)	0.0117 (16)	0.0142 (18)	0.0025 (14)	0.0014 (15)	−0.0007 (15)
C7	0.0168 (18)	0.0191 (19)	0.0120 (19)	−0.0018 (15)	−0.0010 (15)	−0.0024 (16)
C8	0.0138 (17)	0.0193 (19)	0.017 (2)	−0.0018 (15)	0.0004 (15)	−0.0007 (16)
C9	0.0137 (17)	0.0189 (18)	0.017 (2)	0.0020 (15)	−0.0012 (15)	0.0046 (16)
C10	0.0186 (19)	0.025 (2)	0.020 (2)	0.0063 (16)	0.0023 (16)	0.0014 (17)
C11	0.0165 (19)	0.039 (3)	0.026 (2)	0.0049 (18)	0.0050 (17)	−0.004 (2)
C12	0.0152 (19)	0.044 (3)	0.029 (3)	−0.0067 (18)	0.0014 (18)	−0.008 (2)
C13	0.0205 (19)	0.020 (2)	0.030 (2)	−0.0053 (17)	0.0003 (18)	−0.0063 (17)
C14	0.0218 (19)	0.0146 (18)	0.019 (2)	0.0011 (15)	0.0018 (17)	−0.0015 (16)
C15	0.0224 (19)	0.0132 (18)	0.023 (2)	0.0021 (15)	−0.0029 (17)	−0.0010 (16)
C16	0.0155 (18)	0.024 (2)	0.017 (2)	0.0015 (15)	0.0018 (15)	0.0019 (16)

### (II) cis-Bis[µ-2-(1,3-benzothiazol-2-yl)phenolato]-κ3N,O:O;κ3O:N,O-bis[fac-tricarbonylrhenium(I)] Geometric parameters (Å, º)

**Table:**

Re01—C14	1.898 (4)	C2—H2	0.9300
Re01—C15	1.904 (4)	C3—C4	1.390 (4)
Re01—C16	1.918 (4)	C3—H3	0.9300
Re01—O1	2.139 (2)	C4—C5	1.377 (4)
Re01—O1^i^	2.166 (2)	C4—H4	0.9300
Re01—N1	2.214 (3)	C5—C6	1.389 (5)
S1—C1	1.726 (4)	C5—H5	0.9300
S1—C7	1.735 (4)	C7—C8	1.450 (5)
O1—C9	1.355 (4)	C8—C13	1.401 (5)
O1—Re01^i^	2.166 (2)	C8—C9	1.404 (5)
O2—C14	1.153 (4)	C9—C10	1.382 (5)
O3—C16	1.158 (5)	C10—C11	1.370 (5)
O4—C15	1.157 (5)	C10—H10	0.9300
N1—C7	1.318 (4)	C11—C12	1.378 (6)
N1—C6	1.393 (4)	C11—H11	0.9300
C1—C6	1.396 (5)	C12—C13	1.377 (5)
C1—C2	1.398 (5)	C12—H12	0.9300
C2—C3	1.380 (5)	C13—H13	0.9300
			
C14—Re01—C15	88.10 (18)	C5—C4—H4	119.4
C14—Re01—C16	88.70 (15)	C3—C4—H4	119.4
C15—Re01—C16	86.45 (14)	C4—C5—C6	119.4 (3)
C14—Re01—O1	95.63 (14)	C4—C5—H5	120.3
C15—Re01—O1	175.25 (12)	C6—C5—H5	120.3
C16—Re01—O1	96.54 (13)	C5—C6—N1	128.0 (3)
C14—Re01—O1^i^	167.74 (13)	C5—C6—C1	118.5 (3)
C15—Re01—O1^i^	104.12 (14)	N1—C6—C1	113.5 (3)
C16—Re01—O1^i^	92.87 (13)	N1—C7—C8	127.5 (3)
O1—Re01—O1^i^	72.12 (12)	N1—C7—S1	114.0 (3)
C14—Re01—N1	92.80 (12)	C8—C7—S1	118.5 (3)
C15—Re01—N1	96.72 (12)	C13—C8—C9	118.4 (3)
C16—Re01—N1	176.53 (14)	C13—C8—C7	119.4 (3)
O1—Re01—N1	80.20 (10)	C9—C8—C7	122.1 (3)
O1^i^—Re01—N1	85.00 (10)	O1—C9—C10	120.1 (3)
C1—S1—C7	90.05 (17)	O1—C9—C8	120.5 (3)
C9—O1—Re01	120.5 (2)	C10—C9—C8	119.4 (3)
C9—O1—Re01^i^	129.6 (2)	C11—C10—C9	121.1 (4)
Re01—O1—Re01^i^	105.77 (11)	C11—C10—H10	119.4
C7—N1—C6	112.3 (3)	C9—C10—H10	119.4
C7—N1—Re01	120.7 (2)	C10—C11—C12	120.3 (4)
C6—N1—Re01	126.7 (2)	C10—C11—H11	119.8
C6—C1—C2	122.6 (3)	C12—C11—H11	119.8
C6—C1—S1	110.2 (3)	C13—C12—C11	119.6 (4)
C2—C1—S1	127.2 (3)	C13—C12—H12	120.2
C3—C2—C1	117.2 (3)	C11—C12—H12	120.2
C3—C2—H2	121.4	C12—C13—C8	121.0 (3)
C1—C2—H2	121.4	C12—C13—H13	119.5
C2—C3—C4	120.9 (3)	C8—C13—H13	119.5
C2—C3—H3	119.6	O2—C14—Re01	177.6 (3)
C4—C3—H3	119.6	O4—C15—Re01	178.7 (3)
C5—C4—C3	121.3 (3)	O3—C16—Re01	178.3 (3)
			
C7—S1—C1—C6	−1.5 (3)	C1—S1—C7—N1	1.0 (3)
C7—S1—C1—C2	177.1 (4)	C1—S1—C7—C8	−179.2 (3)
C6—C1—C2—C3	−2.7 (6)	N1—C7—C8—C13	−158.5 (4)
S1—C1—C2—C3	178.8 (3)	S1—C7—C8—C13	21.7 (5)
C1—C2—C3—C4	0.0 (6)	N1—C7—C8—C9	25.2 (6)
C2—C3—C4—C5	1.2 (6)	S1—C7—C8—C9	−154.6 (3)
C3—C4—C5—C6	0.2 (6)	Re01—O1—C9—C10	135.2 (3)
C4—C5—C6—N1	178.4 (4)	Re01^i^—O1—C9—C10	−71.1 (4)
C4—C5—C6—C1	−2.8 (5)	Re01—O1—C9—C8	−45.7 (4)
C7—N1—C6—C5	177.8 (4)	Re01^i^—O1—C9—C8	108.0 (3)
Re01—N1—C6—C5	−9.0 (5)	C13—C8—C9—O1	179.7 (3)
C7—N1—C6—C1	−1.0 (4)	C7—C8—C9—O1	−4.0 (5)
Re01—N1—C6—C1	172.2 (2)	C13—C8—C9—C10	−1.2 (5)
C2—C1—C6—C5	4.2 (6)	C7—C8—C9—C10	175.1 (3)
S1—C1—C6—C5	−177.2 (3)	O1—C9—C10—C11	179.5 (3)
C2—C1—C6—N1	−176.9 (3)	C8—C9—C10—C11	0.4 (6)
S1—C1—C6—N1	1.8 (4)	C9—C10—C11—C12	1.1 (6)
C6—N1—C7—C8	−180.0 (4)	C10—C11—C12—C13	−1.8 (6)
Re01—N1—C7—C8	6.3 (5)	C11—C12—C13—C8	0.9 (6)
C6—N1—C7—S1	−0.2 (4)	C9—C8—C13—C12	0.5 (6)
Re01—N1—C7—S1	−173.91 (16)	C7—C8—C13—C12	−175.9 (4)

Symmetry code: (i) −*x*, *y*, −*z*+1/2.

### (II) cis-Bis[µ-2-(1,3-benzothiazol-2-yl)phenolato]-κ3N,O:O;κ3O:N,O-bis[fac-tricarbonylrhenium(I)] Hydrogen-bond geometry (Å, º)

**Table:**

*D*—H···*A*	*D*—H	H···*A*	*D*···*A*	*D*—H···*A*
C4—H4···O2^ii^	0.93	2.49	3.387 (4)	163
C2—H2···O3^iii^	0.93	2.64	3.467 (5)	149

Symmetry codes: (ii) *x*−1/2, −*y*+1/2, −*z*+1; (iii) *x*, *y*+1, *z*.
